# Vascular Calcification: Is it rather a Stem/Progenitor Cells Driven Phenomenon?

**DOI:** 10.3389/fbioe.2018.00010

**Published:** 2018-02-09

**Authors:** Aleksandra Leszczynska, J. Mary Murphy

**Affiliations:** ^1^Regenerative Medicine Institute, Cedars-Sinai Medical Center, Los Angeles, CA, United States; ^2^Regenerative Medicine Institute, National University of Ireland Galway, Galway, Ireland

**Keywords:** vascular calcification, stem cells, atherosclerosis, pericytes, progenitor cells

## Abstract

Vascular calcification (VC) has witnessed a surge of interest. Vasculature is virtually an omnipresent organ and has a notably high capacity for repair throughout embryonic and adult life. Of the vascular diseases, atherosclerosis is a leading cause of morbidity and mortality on account of ectopic cartilage and bone formation. Despite the identification of a number of risk factors, all the current theories explaining pathogenesis of VC in atherosclerosis are far from complete. The most widely accepted response to injury theory and smooth muscle transdifferentiation to explain the VC observed in atherosclerosis is being challenged. Recent focus on circulating and resident progenitor cells in the vasculature and their role in atherogenesis and VC has been the driving force behind this review. This review discusses intrinsic cellular players contributing to fate determination of cells and tissues to form ectopic cartilage and bone formation.

## Introduction

Vascular calcification (VC) is an important complication of atherosclerosis contributing to cardiovascular morbidity and mortality (Alexopoulos and Raggi, 2009), given the increased risk of heart attack with calcified coronaries and the growing incidence of calcified aortic stenosis (Rajamannan et al., 2007). It is increasingly being accepted that VC is far from a passive degenerative process as thought for the last few decades. Rather, the most recent concept is that VC is an active, organized, complex, and highly regulated process reflecting the plasticity of vasculature (Bostrom, 2016). In particular, calcification of atherosclerotic plaque recapitulates virtually the same biologic reactions inherent to normal physiologic bone formation (Neven and D’Haese, 2011). This recapitulation is evidenced by the presence of bone-like structures in the atherosclerotic arteries and valves, which in many cases is structurally complete trabecular bone (Hunt et al., 2002). This resemblance is not just at the macroscopic level but even at microscopic level shows features such as completely formed marrow cavities with hematopoietic and marrow stromal cells (Bunting, 1906; Soor et al., 2008). Energy dispersive X-ray analysis has shown that the mineral in vascular lesions is hydroxyapatite (Ewence et al., 2008) the same mineral as in bone, not just amorphous calcium phosphate.

Inflammation has been shown to be a contributing factor (Bessueille and Magne, 2015). The expression of growth factors, matrix proteins, and other bone-related proteins that are involved in both the initiation and inhibition of mineralization supports the dogma of VC being a cell controlled event (Dallas and Bonewald, 2010). Calcifying vascular cells (CVCs), a subpopulation of vascular smooth muscle cells (VSMCs), that form bone-like calcifying nodules, have been found (Watson et al., 1994; Ting et al., 2011). In fact, the likelihood for the presence of a number of stem progenitor niches and/or lineages in vasculature has been described (Martinez-Agosto et al., 2007; Bautch, 2011). Therefore, despite the fact that the understanding of these resident stem progenitor populations is currently at an early stage, tantalizing prospects for their biological and pathological role are being envisaged. This review focuses on different resident and circulating cells that play a role in ectopic cartilage and bone formation in the vessel wall.

## Progenitor Stem Cells in VC

A number of cell types have been implicated in VC (Table 1). The cells in all three layers of the vessel wall (Media, Intima, and Adventitia) respond to cues in local microenvironment and undergo chondrogenic/osteogenic differentiation. Along with these resident cells, circulating cells have been shown to migrate into the vessel wall and contribute to VC.

**Table 1:** Potential cell types in vascular calcification.

Cell types	Source/location	Ref.
SMCs	Resident	Speer et al. (2009), Tintut et al. (1998), and Speer et al. (2009)
SMC progenitors	Resident	Kramann et al. (2016) and Bardeesi et al. (2017)
Endothelial progenitors	Resident	Wirrig and Yutzey (2014) and Yao et al. (2015)
Circulating stem cells progenitors	Circulating	Cho et al. (2013) and Hu et al. (2003)
HSCs	Circulating	Cianciolo et al. (2016), Nakahara et al. (2017), and Sata et al. (2002)
MSCs	Circulating	Liao et al. (2012)
Pericytes	Resident	Canfield et al. (2000), Davaine et al. (2014), Bardeesi et al. (2017), and Kirton et al. (2006)
Adventitial cells	Resident	Tigges et al. (2013), Kramann et al. (2016), and Bussolati et al. (2017)

### Smooth Muscle Progenitor Cells (SMPCs) and Smooth Muscle Cells (SMCs)

Smooth muscle cell proliferation and matrix protein synthesis, including collagen, elastin, and/or proteoglycans, lead to plaque accretion (Bentzon et al., 2006, 2007) making fibrous tissue a major component of plaque (Cappendijk et al., 2005) and may contribute significantly to coronary artery stenosis (Kataoka et al., 2009). The risk of plaque rupture and a subsequent thrombotic event increases substantially with scarcity of SMCs in the fibrous cap (Schwartz et al., 2000). They also play a role in healing the ruptured plaque by secretion of ECM (Mann and Davies, 1999; Bentzon et al., 2007). However, this healing may result in increased plaque size causing further stenosis (Mann and Davies, 1999; Burke et al., 2001). SMCs found in plaque may be sourced from locally available preexisting SMCs which migrate to the outer layer of plaque (Hu et al., 2004). The SMCs, present in media and intima of the vessel wall, but more importantly cells from the media (Zoll et al., 2008), may form the fibrous component of the plaque while acquiring a synthetic and migrating phenotype through a process called phenotypic modulation (Hao et al., 2006).

This hypothesis that SMC recruitment and phenotypic modulation is being challenged with suggestions that differentiation of progenitor cells such as hematopoietic stem cells (HSCs) contributes to pathogenesis of atherosclerosis (Sata et al., 2002). Also, another possibility suggests that migration of progenitor cells from adventitia contributes to atherosclerotic plaque. Bone marrow-derived circulating progenitor cells have also been claimed as the source of a sizeable proportion of SMCs found in the atherosclerotic lesion (Tanaka et al., 2008). According to some researchers, the phenotypical differences between contractile SMCs with an abundance of myofilaments and synthetic SMCs with plenty of rough ER and golgi complexes do not suggest phenotypic modulation but rather hint toward different sources, such as a subpopulation in the arterial media with a synthetic phenotype (Frid et al., 1994), stem cells in adventitia (Hu et al., 2004; Torsney et al., 2007) or circulating SMPCs (Saiura et al., 2001). In the case of plaque rupture, local SMCs have been reported to have short telomeres and other markers of senescence (Matthews et al., 2006; Minamino and Komuro, 2008), thus suggesting that rupture healing SMCs are not locally proliferating SMCs and may very well be derived from circulating progenitor cells. This is also suggested by the fact that circulating cells can be induced to express SMC proteins like α-smooth muscle actin (α-SMA) or smooth muscle myosin heavy chain. Furthermore, studies have showed that mesenchymal stem cells (MSCs) express α-SMA protein and so also do stem cells derived from the arterial wall (da Silva Meirelles et al., 2006; Klein et al., 2011). The role of SMPCs in atherosclerosis is more complicated. The severity of luminal stenosis has been related to SMPCs, whereas a decrease in SMPC number may be involved in causing a thinner neointima and unstable plaque.

Thus, over the years, research investigating the origin of SMCs in atherosclerotic lesions has swung from an underlying medial origin to circulating progenitor cells of bone marrow origin. However, there are reports with detailed studies showing that the contribution of circulating bone marrow-derived cells to intimal tissue is less likely (Hu et al., 2002; Hillebrands et al., 2003; Daniel et al., 2010). In one study, the authors investigated the origin of SMCs at the healed plaque rupture sites and showed that SMCs healing the plaque ruptures originate from the local vascular wall (Bentzon et al., 2007).

### Endothelial Progenitor Stem Cells and Circulating Progenitor Stem Cells

The functional integrity of the endothelial layer to prevent atherogenic processes is crucial. The known risk factors of coronary artery disease have been shown to induce apoptosis in endothelial cells (ECs), leading to disruption of monolayer integrity (Rossig et al., 2001; Urbich and Dimmeler, 2004b). In atherosclerosis, activation of the damaged ECs triggers the development of the lesions (Zampetaki et al., 2008). Increasingly, it is being understood that endothelial progenitor cells (EPCs) have a strong role in vascular repair by contributing to regeneration of the injured endothelial layer. A negative correlation between severity of atherosclerosis and number of EPCs in patients has been established and an increased number of EPCs has been reported to decrease risk of stroke. EPCs have been described to express CD34, CD133, or vascular endothelial growth factor receptor 2. Of the multiple precursors, hemangioblasts, bone marrow-derived monocytic cells, or tissue resident stem cells are prominent (Zampetaki et al., 2008).

Studies in mice demonstrated that circulating EPCs are directly incorporated in the vessel wall and are involved in re-endothelialization (Zampetaki et al., 2008). A study has shown that the newly regenerated ECs in a model of transplant atherosclerosis were derived from circulating blood from the recipient and not from donor vessels (Hu et al., 2003). In fact, disease development can be prevented by treatment with bone marrow-derived progenitor cells from young non-atherosclerotic ApoE^−/−^ mice in aged ApoE^−/−^ mice (Rauscher et al., 2003). A number of studies have established a direct link between number of EPCs and endothelial repair and reduction in neointima formation (Griese et al., 2003; Wassmann et al., 2006).

However, the new vessel formation supported by these progenitor cells may prove to be detrimental as shown by a study using a hind limb ischemia model in ApoE^−/−^ mice which demonstrated increased plaque size along with improved blood supply to the ischemic areas (Silvestre et al., 2003). Another study observed that EPC treatment led to increased instability of the plaque which can be attributed to their pro-inflammatory effects with a finding of reduced local IL-10 levels. Thus, it seems that EPCs can have opposite effects; impaired mobilization of EPCs may hamper re-endothelialization while excessive mobilization may cause stenosis (Inoue et al., 2007). However, while evaluating the effects of EPCs, their heterogenicity must be considered as different isolation protocols have been shown to affect the functionality of the cells (Seeger et al., 2007). The role of microvessels in the vessel wall in atherogenesis has been emphasized (Kahlon et al., 1992; Moulton et al., 1999; Ross et al., 2001); however, it has also been demonstrated that ECs in these microvessels are derived from progenitor cells (Hu et al., 2003). Thus, considering their role in endothelial repair and plaque angiogenesis, it is clear that EPCs play both beneficial and detrimental roles, at least in transplant atherosclerosis.

### Hematopoietic Stem Cells

Hematopoietic stem cells reside in the arterial tissue and are believed to be involved in maintaining the vascular system on account of their capacity for self-renewal and differentiation to multiple lineages (Feng et al., 2012). Recruitment of circulating blood leukocytes in the vessel wall has been implicated in the development of atherosclerotic plaque (Lusis, 2000; Binder et al., 2002). The role of HSCs in atherosclerosis was investigated recently by inactivating p27 which resulted in enhanced HSC proliferation in arterial macrophages an inflammatory response and accelerated atherosclerosis (Diez-Juan et al., 2004).

### Mesenchymal Stem Cells

Plasticity of MSCs shows their ability to differentiate into several cell types (Narcisi et al., 2015) and is influenced by milieu they are presented with. It has been shown that when primed with chondrogenic factors mimicking the cellular niche present in endochondral ossification, mineralization, and vascularization by MSCs is promoted (Freeman et al., 2016). Processes similar to endochondral or intramembranous ossification also occur in the vascular wall (Neven et al., 2011). In addition, MSCs from bone marrow and resident in the vessel wall, have been demonstrated to differentiate into ECs and SMCs, respectively (Urbich and Dimmeler, 2004a; Torsney et al., 2005).

Circulating MSCs can migrate through the blood stream and reach the site of injury in the vessel wall (Abedin et al., 2004). It is now believed that previously described CVCs in the arterial wall are a type of MSCs, one generation below in mesenchymal hierarchy as suggested by their self-renewal and pluripotent plasticity with lack of adipogenic lineage (Tintut et al., 2003). Regarding the differentiation of MSCs to SMCs, the controversy still remains although, through studies investigating the pathogenesis of transplant arteriosclerosis, the role of MSCs or EPCs in repair of ECs is quite clear along with the apparent role of vascular stem cells in replenishing dead cells (Xu, 2008).

In a study with balloon injury in hyperlipidemic rats, bone marrow-derived MSCs (BM-MSCs) were found to clearly increase the size of atherosclerotic lesions (Liao et al., 2012). In this study, transferring BM-MSCs resulted in VC in medial layers detected at 6 weeks after balloon angioplasty (Liao et al., 2012). Although the exact mechanism of calcification following BM-MSCs transfer is not clear, there was a possible association with upregulation of BMP-2 (Nakagawa et al., 2010; Liao et al., 2012). Evidence suggests that the regulation of this calcification involves locally expressed bone calcification regulatory factors (Hruska et al., 2005; Johnson et al., 2006; Ketteler et al., 2006). Another study has demonstrated that transfer of bone marrow and EPCs not only stimulate disease progression in atherosclerosis but also impacted stability of the plaque (George et al., 2005). Studies have provided enough evidence to suggest a link between MSC transfer and pathogenesis of atherosclerosis (Saiura et al., 2001; Sata et al., 2002).

### Vascular Stem Cells

There is evidence that stem or progenitor cells reside in different organs and differentiate to repair the injury. However, it might be that these resident stem/progenitor cells are not only contributing to the repair but play an important role to the pathogenesis of atherosclerosis (Kawabe and Hasebe, 2014; Yu et al., 2017). A number of cell types present in vessel wall have been shown to potentially be the vascular stem cells (Tilki et al., 2009; Bautch, 2011; Psaltis et al., 2011; Torsney and Xu, 2011; Bostrom et al., 2012). By far, pericytes are the most important VSCs and will be discussed further.

### Pericytes

Pericytes are elongated cells, around 70 µm long, embedded within the basement membrane adjacent to the EC junctions (Voisin et al., 2010; Proebstl et al., 2012). Being closely associated with endothelium, they play a crucial function in maintaining vessel wall integrity and contributing to the generation of the venular basement membrane (Armulik et al., 2005; Edelman et al., 2006). Pericytes have been identified in the inner intimal layer and also the outer layer of the media in vasa vasora in adventitia of large, medium, or small arteries and veins.

It has been suggested that pericytes are MSCs associated with the blood vessel walls where they serve as a support to these vessels (Caplan, 2008, 2017; Crisan et al., 2009). These cells can differentiate not only into fibroblasts, SMC but also into different lineages, especially to osteoblasts (Canfield et al., 1996; Collett and Canfield, 2005; Klein et al., 2011), chondrocytes (Schor et al., 1990; Farrington-Rock et al., 2004; Klein et al., 2011), or adipocytes (Farrington-Rock et al., 2004; Klein et al., 2011) under appropriate cell culture conditions. Pericytes can also act as macrophage precursors and express macrophage markers such as CD4 class I and class II MHC molecules and perform macrophage-like activities (Bergers and Song, 2005; Hall, 2006).

The heterogeneity of pericytes is reflected in their function (Schor et al., 1990). In large arteries, pericytes are embedded within the endothelial basement membrane. They facilitate and integrate cell communication (Armulik et al., 2005). Usually, pericytes overlap several ECs and regulate certain functions by secretion of factors (Armulik et al., 2005; Thanabalasundaram et al., 2011). Pericytes extend long processes exhibiting contractile microfilament bundles which wrap around the blood vessel (Dore-Duffy and Cleary, 2011). They can also form more confined finger like projections and retract them when migrating (Dore-Duffy and Cleary, 2011).

Pericyte coverage varies considerably in different organs, implying their varied functions in different tissues (Proebstl et al., 2012). Similar to VSMCs, pericytes are thought to have multiple origins. In the axial and lateral plate mesenchyme, the vessel wall cells around the developing trunk vessels have been attributed to a mesodermal origin (Hungerford and Little, 1999). In the central nervous system, they might also be derived from neurocrest (Etchevers et al., 2002; Bergers and Song, 2005), at least partly (Etchevers et al., 2001), or mesodermal precursors called angioblasts (Carmeliet, 2004). On the other hand, coronary vessel wall cells have been thought to develop from epicardial cells which have a splanchnic mesodermal origin (Gittenberger-de Groot et al., 1999; Vrancken Peeters et al., 1999). Nevertheless, in general, pericytes are considered to be of mesenchymal origin. They are thought to be associated with MSCs (Creazzo et al., 1998; Armulik et al., 2005; Lamagna and Bergers, 2006) since they can differentiate into different cell types. A study investigating adult angiogenesis confirmed the bone marrow origin of mural cells (Rajantie et al., 2004). Though not a major pathway of pericyte formation in normal physiological development, there has been some evidence of transdifferentiation from ECs (DeRuiter et al., 1997; Rajantie et al., 2004; Armulik et al., 2005), where TGF-β3 can initiate the differentiation.

**Figure 1:**
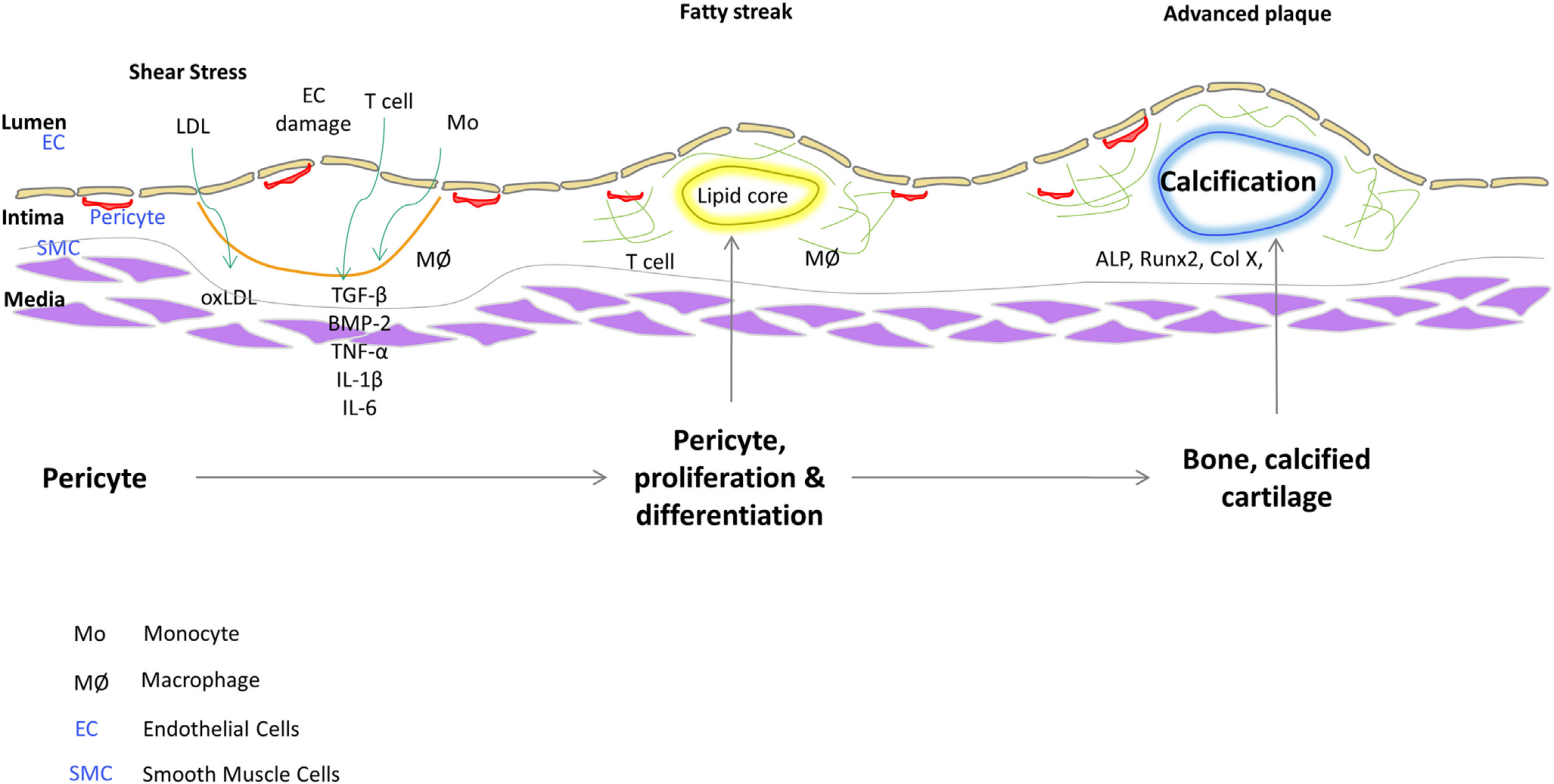
Plausible role of vascular stem cells/pericytes in progression of atherosclerotic plaque.

Pericytes are distinguished from other cell types by their marker expression. They express different markers during the different stages of their growth and also depending on their origin. Pericytes are clearly distinguished from other stromal cells such as SMCs and ECs; however, they share many similarities with other cells like myofibroblasts (Alexander et al., 2005). There are several markers which are used to characterize pericytes like α-SMA (Morikawa et al., 2002; Song et al., 2005), NG2 (Ozerdem et al., 2002; Song et al., 2005; Klein et al., 2011) (nerve/glial antigen 2), PDGFR-β (Winkler et al., 2010; Klein et al., 2011), aminopeptidase A (Ozerdem and Stallcup, 2003), and RGS5 (Gerhardt and Betsholtz, 2003) (regulator of G-protein signaling 5). It is believed that none of the markers absolutely characterize the pericytes on account of their different origins and stage of development. Some of the markers might be expressed dynamically and vary between different organs. 3G5, considered to be a pericyte-specific marker is found on the surface of the pericytes (Nayak et al., 1988; Juchem et al., 2010). 3G5 is expressed in pericytes of human and bovine aorta (Bostrom et al., 1993; Juchem et al., 2010). The role of pericytes as cellular models of atherogenesis has recently been reviewed (Ivanova and Orekhov, 2016). Figure 1 shows a schematic that illustrates a role of vascular stem cells or pericytes in atherosclerotic plaque progression based on our findings. In fact, we have described a population of pericyte-like progenitor cells isolated from aortae of ApoE^−/−^ mice and control C57BL/6 mice and shown their possible role in aberrant tissue formation in the atheromatous plaque (Leszczynska et al., 2016).

As we continue to unravel the mysteries underlying disease progression in atherosclerosis and decipher the role of the contributing stem/progenitors in VC, the importance of understanding the cell–cell and cell–tissue communication, especially through the microvesicular/exosomal pathway, cannot be overemphasized. In fact, many studies in recent years have shown important role played by exosomes in communication protocol in atherosclerosis (Huber and Holvoet, 2015; Gao et al., 2016; Perrotta and Aquila, 2016; Lu, 2017).

## Conclusion

A number of stem progenitor cells are involved in VC, in fact they are emerging as the most important players. On account of their plasticity and involvement in VC and inflammation, pericytes are arguably the most interesting ones among the different contributors. Going forward, it will be increasingly important to understand how exosomes execute the bio-message delivery among different key players and exploit therapeutic potential thereof.

## Author Contributions

AL and JMM conceived the idea of this article. AL wrote this mini review and JMM revised and finalized it.

## Conflict of Interest Statement

The authors declare that the research was conducted in the absence of any commercial or financial relationships that could be construed as a potential conflict of interest.
